# Exploring the Role of Phenylalanine Residues in Modulating the Flexibility and Topography of the Active Site in the Peroxygenase Variant PaDa-I

**DOI:** 10.3390/ijms21165734

**Published:** 2020-08-10

**Authors:** Joaquin Ramirez-Ramirez, Javier Martin-Diaz, Nina Pastor, Miguel Alcalde, Marcela Ayala

**Affiliations:** 1 Departamento de Ingeniería Celular y Biocatálisis, Instituto de Biotecnología, Universidad Nacional Autónoma de México, Av. Universidad 2001 Col. Chamilpa, Cuernavaca 62210, Morelos, Mexico; joako@ibt.unam.mx; 2 Department of Biocatalysis, Institute of Catalysis and Petrochemistry, CSIC, 28049 Madrid, Spain; javiermd44@hotmail.com; 3 Centro de Investigación en Dinámica Celular, IICBA, Universidad Autónoma del Estado de Morelos, Cuernavaca 62209, Morelos, Mexico; nina@uaem.mx

**Keywords:** biocatalysis, molecular dynamics, oxizyme engineering, peroxygenases, structure–function relationship

## Abstract

Unspecific peroxygenases (UPOs) are fungal heme-thiolate enzymes able to catalyze a wide range of oxidation reactions, such as peroxidase-like, catalase-like, haloperoxidase-like, and, most interestingly, cytochrome P450-like. One of the most outstanding properties of these enzymes is the ability to catalyze the oxidation a wide range of organic substrates (both aromatic and aliphatic) through cytochrome P450-like reactions (the so-called peroxygenase activity), which involves the insertion of an oxygen atom from hydrogen peroxide. To catalyze this reaction, the substrate must access a channel connecting the bulk solution to the heme group. The composition, shape, and flexibility of this channel surely modulate the catalytic ability of the enzymes in this family. In order to gain an understanding of the role of the residues comprising the channel, mutants derived from PaDa-I, a laboratory-evolved UPO variant from *Agrocybe aegerita*, were obtained. The two phenylalanine residues at the surface of the channel, which regulate the traffic towards the heme active site, were mutated by less bulky residues (alanine and leucine). The mutants were experimentally characterized, and computational studies (i.e., molecular dynamics (MD)) were performed. The results suggest that these residues are necessary to reduce the flexibility of the region and maintain the topography of the channel.

## 1. Introduction

The unspecific peroxygenase (UPO, E.C. 1.11.2.1) is a versatile heme-thiolate oxidoreductase capable of catalyzing the oxidation of a wide range of organic substrates using hydrogen peroxide as oxidizing agent [1,2,3]. The most remarkable feature of this fungal enzyme is the hydroxylating activity towards non-activated aromatic and aliphatic hydrocarbons without the need of other cofactors, such as NAD(P)H, or accessory protein domains [4,5,6]. Thus, UPO is a promising tool for biotechnological applications in organic synthesis and bioremediation [7,8,9]. The first UPO was described in the basidiomycete *Agrocybe aegerita* [1], followed by UPO from *Coprinus radians* [10], *Marasmius rotula* [11], and *Chaetomium globosum* [12], among others. To date, there are hundreds of putative peroxygenase sequences identified in fungal genomes and even the long-time peroxidase outsider *Leptoxyphium fumago* chloroperoxidase (LfuCPO) has been catalogued as a peroxygenase, albeit specialized in halide oxidation [13,14]. In UPOs, there is a channel connecting the bulk solvent with the active site that allows substrate access to the heme group, and thus facilitates two-electron oxidations through oxygen atom transfer (peroxygenase activity) [15,16,17,18,19]. The size and shape of the heme channel vary depending on the peroxygenase. For example, in LfuCPO, the first heme-thiolate peroxidase described back in the 1960s [20], the channel has a maximum width of 7.2 Å, whereas in *Agrocybe aegerita* UPO (AaeUPO) its width is 9.8 Å [15], and in *Marasmius rotula* UPO (MroUPO) it is shorter but wider than in AaeUPO (around 12.9 Å) [18]. These features, along with the different amino acids upholstering the channel (mostly aromatic residues for AaeUPO and aliphatic ones for MroUPO), are proposed as key determinants in the extraordinary versatility of UPOs [15,16].

Understanding the molecular factors that modulate the oxidative capacity of these enzymes is fundamental for the design of improved biocatalysts with biotechnological potential [20,21]. Computational tools are increasingly used in protein engineering, combined with experimental approaches such as directed evolution and/or site-directed mutagenesis [22]. Protein simulation allows to predict the effect of site-directed mutagenesis as well as to explain experimental results. In the case of UPOs, computational tools such as Protein Energy Landscape Exploration (PELE) and mixed quantum mechanics/molecular mechanics (QM/MM) have been used to explain the enhanced activity towards naphthalene of AaeUPO variants obtained by directed evolution [23] and to explain the differences observed in the steroid hydroxylation by basidiomycete peroxygenases [8]. Furthermore, PELE combined with molecular dynamics (MD) and QM/MM simulations were used to obtain structural insights into the changes caused by a single amino acid mutation in evolved AaeUPO variants obtained for the selective hydroxylation of propranolol [24]. 

In this work, we explored the topography of the substrate access channel by combining computational simulations and site-directed mutagenesis. We used as a model the recombinant variant of the AaeUPO (PaDa-I) previously obtained by directed evolution [25] and standard substrates as well as polycyclic aromatic hydrocarbons (PAHs) as models of bulky molecules, to characterize the generated variants [26]. Molecular dynamics helped to shed light on the role in protein flexibility of residues conforming the channel, while site-directed mutagenesis of specific phenylalanine residues led to a better understanding of the role of channel composition in the catalytic properties of the enzyme.

## 2. Results and Discussion

PaDa-I is an AaeUPO variant obtained through directed evolution, which contains amino acid replacements that enhance its recombinant expression in yeast and is thus amenable to protein engineering. The mutations in PaDa-I are at least 10 Å away from the active site and none is localized directly in the substrate access channel; it has been reported that these mutations do not affect the overall catalytic behavior of the enzyme compared to the wild-type AaeUPO [25].

**Figure 1:**
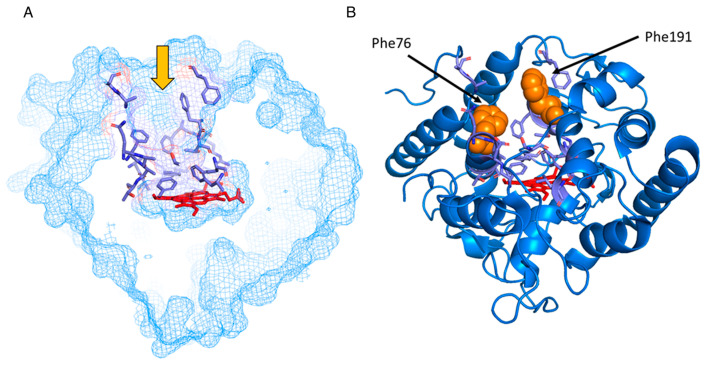
(**A**) Substrate access channel in PaDa-I model structure (PDB ID: 5OXU). The topography of the channel is delimited by the following residues (in violet): Phe69, Asp70, Gln72, Ala73, Phe76, Ala77, Thr78, Ala80, Ala81, Phe121, Phe188, Arg189, Phe191, Thr192, Glu196, Phe199, Leu203, Ser240, Phe274, Ala316, and Ala317. The orange arrow signals the entrance to the channel that ends in the heme pocket (heme group in red). (**B**) Residues Phe76 and Phe191 (light orange) in the substrate access channel were replaced with Ala or Leu in variants F76A, F191A, F76L, F76A/F191A, and F76L/F191A.

Visual inspection of the crystallographic structure of PaDa-I (PDB ID: 5OXU) allowed the identification of a channel connecting the distal side of the heme prosthetic group to the bulk solvent (Figure 1). Among the residues conforming this channel, two phenylalanine residues can be clearly identified that orient their side chain moiety towards the channel, namely Phe76 and Phe191, located 10 Å and 14 Å away from the heme iron, respectively (Figure 1). It has been suggested that these residues regulate the entrance of substrates to the heme channel and may impose an upper limit to the size of substrates that can access the active site [15]. Thus, bulky substrates, such as the PAHs pentacene and fluorene, are not oxidized by AaeUPO, probably because they cannot be accommodated close to the heme group, according to molecular docking simulations [15]. According to previously reported crystallographic data, the residue Phe191 is more flexible in the PaDa-I variant, with respect to the wild-type AaeUPO, due to the effect of the mutation F311L; apparently, the smaller side chain in Leu displaces the side chain in Phe76 and this weakens the interaction with Phe191 [16]. However, as mentioned before, this has no effect on the catalytic behavior of the PaDa-I variant.

In order to experimentally characterize the role of these phenylalanine residues, PaDa-I and the variants F76A, F191A, and F76A/F191A were designed to evaluate the effect of a reduced side chain. Preliminary in silico modeling of the variants showed that mutation F76A generates an extra cavity in the heme channel. Therefore, the variants F76L and F76L/F191A were additionally considered to study the effect of a less drastic reduction of the side chain size. All the variants were heterologously produced in *Saccharomyces cerevisiae*. All the variants were active and were functionally expressed—secreted by yeast—at similar total protein levels, as previously reported [25]. As each variant showed a different catalytic behavior (Table 1), the Reinheitszahl value (Rz, a specific parameter for heme proteins that relates the absorbance ratio between 420 nm (Soret band) [2] and 280 nm) was preferred over the specific activity as purity criterion. The purified variants showed an Rz value of ~2.0, which was expected for a homogeneous sample [25]. Additionally, selected variants with an Rz of ~2.0 were analyzed by SDS-PAGE to confirm the presence of a single band (Figure S1, Supplementary Materials). Thus, routine purifications relied on the Rz value as a standard for purity. 

Kinetic characterization of the mutants was performed using 5-nitro-1,3-benzodioxole (NBD) as standard colorimetric assay for peroxygenase activity and naphthalene as model for more complex aromatic substrates (Table 1; Figures S2–S4, Supplementary Materials). Regarding the oxidation of NBD by all mutants, similar or slightly higher (1.6-fold) k_cat_ values were observed. However, in the case of the F76A and double mutant variants, an approximately 2-fold increase in Km for the relatively small NBD molecule was observed, resulting in a 40–50% decrease of k_cat_/Km. On the contrary, for F191A and F76L variants a similar or slightly lower Km (−30%) was observed, resulting in a 30% and 36% increase of k_cat_/Km, respectively. For another aromatic and bulkier substrate such as naphthalene, a modest decrease in catalytic efficiencies was observed for F76L, F191A, and F76L/F191A variants, whereas a larger effect was observed for variants containing the F76A mutation, of up to a 2-fold decrease of k_cat_/Km. It is clear that the replacement of F76 exerts a negative effect on the catalytic efficiency of the enzyme, specifically on substrate affinity, which is more intense if replaced by a small, simple residue such as alanine, whereas the replacement by a larger residue such as leucine buffers this effect. However, the replacement of F191 may be beneficial for the oxidation of the small NBD but reduces the affinity for the bulkier naphthalene. Thus, the elimination of these two bulky aromatic residues apparently reshapes the topography of the channel in a way that may reduce the affinity for some complex substrates.

It has been proposed that peroxidase activity (one-electron oxidation) occurs in the periphery of the protein, independently of direct substrate–heme interaction [27]. Thus, kinetic characterization with ABTS, a typical peroxidative substrate, was also performed for PaDa-I and the variants (Table 1). In this case, the effect of the mutations appears to be more uniform. Variants containing the F76A mutation showed a decreased catalytic efficiency, mainly due to a decrease in k_cat_, whereas variants containing the F191A mutation and/or F76L mutation showed a slightly higher catalytic efficiency, with the larger effect observed for the F76L/F191A variant, in which a 2-fold increase in k_cat_/Km was observed. Unexpectedly, the positive effect in *k*_cat_/Km was mainly due to a decrease in the Km value.

**Figure 2:**
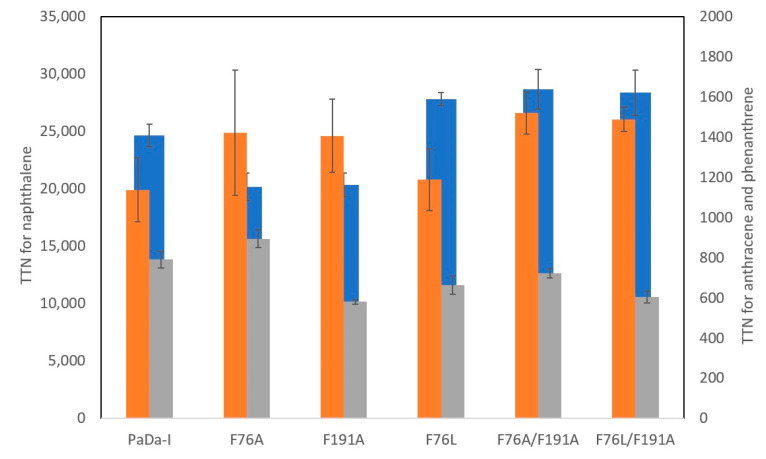
Total turnover number (TTN) of PaDa-I and all the variants with PAHs: naphthalene (blue), anthracene (orange), and phenanthrene (gray). TTN is expressed as moles of substrate converted per mole of enzyme used.

To gain a better understanding on the effect of reshaping the substrate access channel, we determined the total turnover number (TTN) for the oxidation of PAHs of increasing size such as anthracene and phenanthrene. Due to solubility limitations, kinetic constants could not be determined for these compounds. However, TTN is an indicator of catalytic effectiveness, which takes into account different phenomena governing the enzyme behavior during the reaction, such as catalytic efficiency but also catalyst stability. It is a dimensionless number (moles of generated product per moles of consumed enzyme) and it relates product yield to catalyst input [28]. As shown in Figure 2, only small differences in TTN are observed for PaDa-I and the variants for naphthalene oxidation; a 20% decrease in TTN was observed for F76A and F191A variants, whereas a 12–16% increase in TTN was observed for F76L, F76A/F191A, and F76L/F191A. No statistically significant difference in TTN for anthracene oxidation was found among the variants. For phenanthrene, all variants showed similar or lower TTNs (up to 30%) than PaDa-I, although the reduction in TTN was statistically significant only for the F191A variant.

One of the factors that could be affecting the activity of the variants is their operational stability. It is well known that heme-peroxidases can be inactivated by hydrogen peroxide if the reducing substrate is not a good electron donor or it is not readily accessible to the heme [29,30,31]. In the case of UPOs, the main inactivation mechanism seems to be heme destruction, due to oxidation by highly oxidizing species such as hydroxyl radicals [31]. We determined the first-order inactivation constants for the enzyme variants in the presence of 1 mM H_2_O_2_ and without a reducing substrate (Table 2; Figure S5, Supplementary Materials). Half-life values illustrate that all the variants are quickly inactivated. However, it is clear that mutations in position 76 lead to a slightly faster inactivation that could be related to the lower catalase activity of the F76/A,L variants (Table 2). A general trend was observed in which the catalase activity was inversely related to the inactivation constant, the exception being the F76L/F191A mutant, which has a high catalase activity, almost 2-fold higher than PaDa-I. 

In order to further identify the role of residues shaping the topography of the active site, molecular dynamics (MD) simulations were performed and the behavior of residues within the channel was analyzed for PaDa-I and the in silico-constructed variants F76A, F76L, and F191A and the double mutants F76A/F191A and F76L/F191A. It was observed that the average root-mean-square deviation (RMSD) of the whole protein for PaDa-I and the mutants showed a similar behavior, with average RMSD ranging from 1.8 to 2.8 Å (Figure S6, Supplementary Materials). Furthermore, the RMSD of the residues that constitute the heme channel showed a similar behavior in all the enzyme variants (Figure S7, Supplementary Materials).

**Figure 3:**
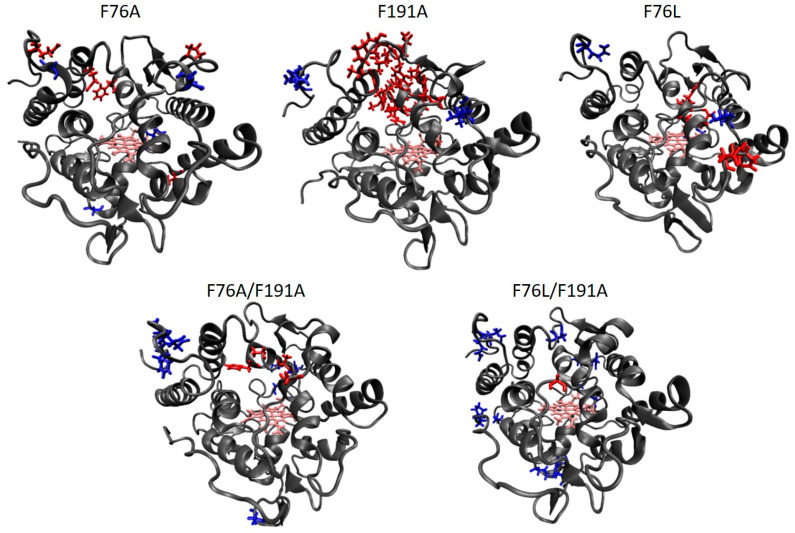
Protein models of the PaDa-I variants showing the localization of the residues with increased (red) and decreased (blue) mobility. The heme group is shown in pink and the protein backbone in a grey ribbon. For the details of residue identity, see Table S2 (Supplementary Materials).

The root mean square fluctuation (RMSF) parameter was used for evaluating the mobility of specific amino acid residues. The RMSF values were calculated for the alpha carbons of each residue in PaDa-I and the variants, and the value obtained in PaDa-I was subtracted from that of the variants. We identified those residues with an RMSF difference greater than one standard deviation of the mean of the differences between PaDa-I and each particular variant. Those residues are considered to have a change in mobility and are shown in Table S1 (Supplementary Materials) and Figure 3.

Variant F76A had only a few changes in mobility in specific residues (Table S1). In contrast, variants F191A and F76L showed an increased mobility in several residues that are part of one of the alpha helixes that constitute the heme channel (from Asp187 to Tyr194 in F191A and from Ala193 to Thr197 in F76L; Table S1, Figure 3). The double mutant F76A/F191A showed some similarities to the F191A variant, with increased mobility in residues Phe188, Ala191, Ala193, and Gly243 (Table S1, Figure 3). Interestingly, the combination of F76L/F191A mutations in the double mutant causes a decrease in mobility in several residues, in regions that are not altered in the other PaDa-I variants. These changes of mobility in the variants could be explained by the reorientation of a network of residues that hold the two alpha helixes together with a loop. In PaDa-I, these residues include Phe76, Met280, Pro277, Phe188, and Phe191, which are in close contact (<5 Å) in a region that could be considered a hot spot for the integrity of the heme channel (Figure S8, Supplementary Materials). The reorientation of this network due to the mutations of Phe76 and Phe191 causes the loss of close interactions between these residues and gives some flexibility to the loop where Met280 is located and to the alpha helix where residue 191 is present (Table S2). Interestingly, in the case of the double mutant F76L/F191A, the reorientation of this network favors a close packaging that does not allow flexibility of the mentioned loop and helix (Figure S8, Table S1). 

**Figure 4:**
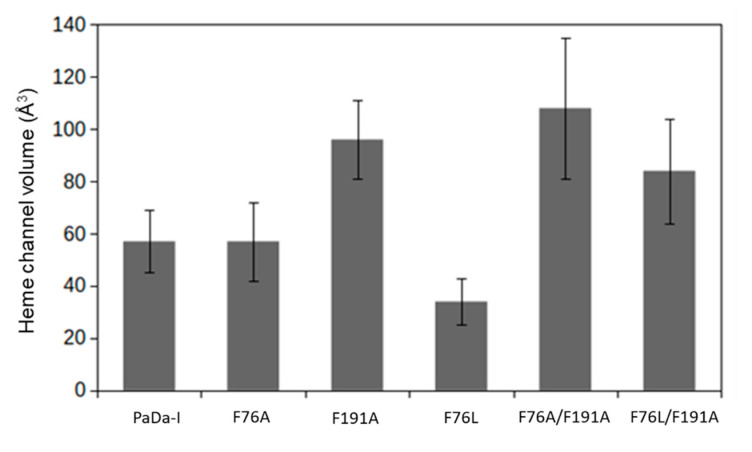
Heme channel volume calculated over the last 100 conformations of the molecular dynamics (MD) simulation.

According to our simulations, the mutations also had an effect on the topography of the heme channel. We calculated the volume of the channel of the last 100 conformations of the MD simulation, which is a representative sample of the system at equilibrium (Figure 4). The volume of the channel did not change in the F76A variant, but it can explore a different topography (see below). F191A and F76A/F191A showed a 70–90% increase in channel volume, whereas the F76L mutation led to a reduced one (Figure 4). 

**Figure 5:**
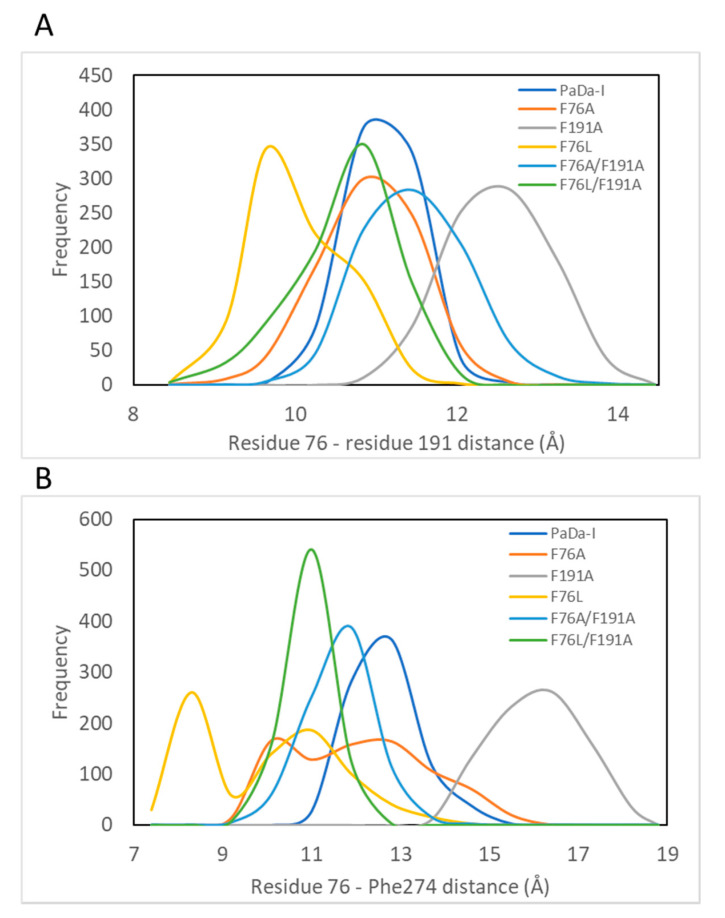
Frequency distribution of distances from residue number 76 to 191 (panel **A**) and from residues 76 to 274 (panel **B**). The distance was measured between the two alpha carbons during the last 40 ns of the MD simulation.

In order to clarify the specific mobility changes that may alter the topography of the heme channel, the arrangement of the loop that contains Phe274 and the helix that harbors the residue in position 191 was analyzed. The most notable arrangement occurs in the F76L variant, where the Phe274 and Phe191 residues move closer to the Leu76 residue (Figure 5); this could explain the reduced channel volume in this variant. On the contrary, a larger channel volume in the F191A variant is the result of a structural rearrangement that is associated with a higher dynamic behavior of the region (Figure 3 and Figure 5); for example, an increased distance between Ala191-Phe274 and Phe76-Ala191 residues is observed (Figure 5). In the case of the F76A/F191A variant, the higher variability of the channel volume (Figure 4) reflects a more dynamic topography as the Phe274-Ala76 distance decreases, whereas the Ala191-Ala76 distance increases (Figure 5). Although in the F76A variant the Phe274 residue explores conformations in which it moves closer to the Ala76 residue, it does not significantly alter the location of Phe191 and Phe274 residues with respect to Ala76, which is in accordance with a heme channel volume similar to PaDa-I, while the double mutant F76L/F191A shows an intermediate behavior between its corresponding single mutants (Figure 5). 

**Figure 6:**
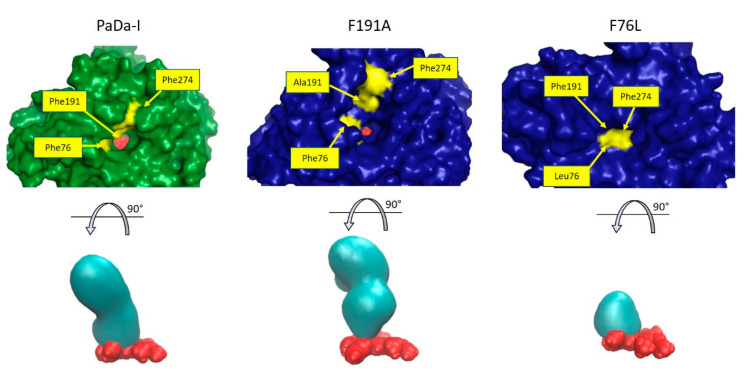
Upper view of the heme channel of the parental PaDa-I (green) and mutants F191A and F76L (blue), showing the changes in topography. The heme group is shown in red spheres and residues 76, 191, and 274 in yellow surface. In the lower part, the shape of the volume (in cyan) conforming the heme channel with respect to the heme group (red spheres) is shown.

In Figure 6, the topography of the cavity formed by residues in the channel is depicted for PaDa-I and the F76L and F191A single mutants; the shape of the cavities for all the variants can be seen in Figure S9 (Supplementary Materials). The changes in topography could be related largely to a rearrangement of the above-mentioned heme channel residues, because the rotational behavior and conformation of the Phe side chains is not altered in the mutants (Figure S10, Supplementary Materials). It is clear that in both mutants the topography of the channel is greatly altered, compared to PaDa-I. While for the F191A variant the cavity defining the channel acquires a more irregular shape, in the F76L variant the cavity is greatly reduced; in both cases, substrate access to the channel could be restricted.

From the analysis of the MD simulations, the Phe76 residue seems to have a role in delimiting the shape of the heme channel; its replacement by simple, smaller residues leads to an altered topography and a greater mobility of some residues of a loop and an alpha helix that shapes the heme channel. In general, these changes are detrimental for the catalytic efficiency, as observed for substrates that must get close to the heme to be hydroxylated (naphthalene and NBD). 

On the contrary, Phe191 has a subtler role that may be related to modulation of the access of specific substrates. In this regard, a recent study on UPO phylogeny, based on genome mining, led to the classification of 113 putative sequences retrieved from 35 different fungal species [13]. In that work, it is proposed that UPOs are divided into five subfamilies, with AaeUPO belonging to subfamily I. According to multiple alignments performed in the mentioned work ([13]), positions 76 and 191 are highly conserved in subfamily I, whereas in the closest sequence-related subfamily (subfamily V) the corresponding positions may be occupied by smaller residues such as Ile in position 76 and Leu in position 191 (Figure S11, Supplementary Materials).

The relationship between topography, composition, and flexibility of the channel (given by certain residues in key positions), and enzyme specificity is still to be elucidated. Only a handful of UPOs have been experimentally characterized, thus a larger number of UPOs from different subfamilies need to be isolated or heterologously expressed in order to deepen our understanding of the catalytic versatility of this group of enzymes.

## 3. Materials and Methods

### 3.1. Computational Methods

#### 3.1.1. System Preparation

The crystal structure of the PaDa-I variant (PDB ID: 5OXU) was used as the starting model [16]. The system was built with the Protein Preparation Wizard of the Maestro 9.8 package (Schrödinger, LCC, New York, NY, USA) [32]. Original water molecules and buffer ions were omitted in the simulations. The protonation state of the amino acid residues at pH 7.0 was established according to the pKa values obtained from the PropKa 3.0 server. All glutamic and aspartic acid residues were deprotonated except for Asp85. Histidine residues were δ-protonated, except for His82 which was ε-protonated and His118 and His251 were doubly protonated. These residues are not located near the substrate access channel (Figure S12, Supplementary Materials). For molecular dynamics simulations, the enzyme ground state was modeled with a heme group containing an Fe(III) cation. PaDa-I-derived variants (F76A, F191A, F76L, F76A/F191A, and F76L/F191A) were generated with the “Build fragments” tool included in Maestro in the ground state. With System Builder, an explicit solvent model with periodic boundary conditions was added (SPC, cubic box shape, 10 Å from the protein surface). The system was neutralized with 4 Na^+^. A minimization of the system was performed with the Desmond software (version 2015.4, Schrödinger, LCC, New York, NY, USA) [33]. The method used was the steepest descent with a convergence threshold of 0.01 kcal/mol/Å. Coulombic interactions were calculated with a cutoff radius of 12 Å. Before the molecular dynamics simulations (Section 3.1.2.), each mutant was minimized with this method after the in silico modeling.

#### 3.1.2. Molecular Dynamics Simulations

Molecular dynamics simulations of the enzymes were performed with the Desmond software [33]. The OPLS_2005 force field was used for the 50 ns simulation in an NPT ensemble. A Berendsen thermostat maintained the temperature at 300 K and the pressure was controlled by the Berendsen algorithm at 1 atm. The RESPA integrator solved the equations of motion (2 fs time step). Short-range interactions had a calculation cutoff of 12 Å, while the electrostatic component was modeled using the particle-mesh Ewald method with a tolerance of 10^−9^. Atom distances and residue movements were analyzed with the VMD molecular visualization program [34]. The heme channel was defined by the following residues in PaDa-I: Phe69, Asp70, Gln72, Ala73, Phe76, Ala77, Thr78, Ala80, Ala81, Phe121, Phe188, Arg189, Phe191, Thr192, Glu196, Phe199, Leu203, Ser240, Phe274, Ala316, and Ala317. For the calculation of the root mean square fluctuation (RMSF), we used the last 40 ns of the MD simulation. This time lapse was further subdivided into packages of 100 ps. In every package, an average structure was calculated for further use as the reference structure. The RMSF was calculated considering only the alpha carbon of the amino acid residues, averaging the RMSFs of all the packages. To identify amino acids with increased or decreased mobility in the enzyme variants with respect to PaDa-I, only those residues that had an RMSF difference greater than one standard deviation of the mean difference were considered. For the calculation of the volume of the heme channel, POVME3.0 (University of California, San Diego, CA, USA) was used [35]. Two inclusion spheres were defined as follows: one was calculated as the center of mass of the triad Phe69, Phe121, and Phe199 (using the ζ-carbons of the side chain), which is close to the heme group; the other one, as the center of mass between the residues 76 and 191 (β-carbon, δ1-carbon, and ζ-carbon were used as reference for Ala, Leu, and Phe, respectively), which are located in the upper part of the heme channel. Both spheres had a radius of 4 Å.

### 3.2. Experimental Section

#### 3.2.1. Site-Directed Mutagenesis

The plasmid pJRoC30-PaDa-I contains the gene for the expression of the laboratory-evolved PaDa-I variant [25]. The enzyme variants were generated by In Vivo Overlap Extension [36]. Specific oligonucleotides were designed to introduce mutations in residues Phe76 and Phe191 (see Table S2). External primers (RMLN and RMLC) with homologous overhangs to the plasmid were used in two separate PCR reactions (Table S2. Reaction 1 contained pJRoC30-PaDa-I as DNA template and RMLC and f76a (or f76l or f191a)-Forward as primers; reaction 2, RMLN and f76a (or f76l or f191a)-Reverse. The PCR protocol consisted of 30 cycles of a 2 min denaturation step at 95 °C, an aligning step of 1 min at 50–55 °C and an extension step of 1 min at 72 °C, and a final extension of 10 min at 72 °C, all catalyzed by Pfu-Polymerase (2.5 U) (Thermo Scientific, Waltham, MA, USA). PCR products were purified with the NucleoSpin Gel and PCR Clean-up Kit (Macherey Nagel, Düren, Germany) and co-transformed with BamHI- and XhoI-linearized plasmid into competent yeast cells using the protocol mentioned in Section 3.2.2. After three days of incubation, single colonies were selected and cultivated in YPD liquid medium for biomass production. The cells were pelleted and the plasmid was extracted using the Zymoprep Yeast Plasmid Miniprep I (Zymo Research, Irvine, CA, USA). Competent *Escherichia coli* XL1-Blue cells were transformed and plasmid was purified by standard protocols. Nucleotide sequences were verified at the “Unidad de Síntesis y Secuenciación de AND” of the Institute of Biotechnology (UNAM). Double mutants were generated by following the same protocol, using the corresponding mutated plasmid as template for the PCR reaction.

#### 3.2.2. Enzyme Production

PaDa-I and the enzyme variants were expressed in *Saccharomyces cerevisiae* BJ5465 as previously reported [25]. The plasmid pJRoC30 (CALTECH, Pasadena, CA, USA) with the PaDa-I gene (or mutant genes) inserted between BamHI and XhoI sites was transformed into *S. cerevisiae* competent cells using the Yeast Transformation Kit (Sigma-Aldrich, St. Louis, MO, USA). Cells were inoculated in agar plates (2%) containing yeast nitrogen base (6.7 g L^−1^), yeast synthetic drop-out medium supplements without uracil (1.92 g L^−1^), glucose (20 g L^−1^), and chloramphenicol (25 mg L^−1^). Plates were incubated at 30 °C for three days. 

Single colonies were selected and inoculated into a 50 mL Erlenmeyer flask with 5 mL of liquid medium containing D-(+)-raffinose (20 g L^−1^), yeast nitrogen base (6.7 g L^−1^), yeast synthetic drop-out medium supplements without uracil (1.92 g L^−1^), and chloramphenicol (25 mg L^−1^). The cultures were incubated for 2 days at 30 °C and 220 rpm and they were used to inoculate a 500 mL flask with 100 mL of medium. Once the optical density at 600 nm reached 1.0, the whole culture was poured into a 2.8 L Fernbach flask with 900 mL of expression medium that contained yeast extract (10 g L^−1^), peptone (20 g L^−1^), potassium phosphate buffer (60 mM, pH 6.0), galactose (20 g L^−1^), magnesium sulfate (1 mM), ethanol (3% *v*/*v*), FeCl_3_ (0.5 mM), and chloramphenicol (25 mg L^−1^). Flasks were incubated for 72 h at 25 °C and 220 rpm. 

#### 3.2.3. Enzyme Purification

Crude extracts were obtained by centrifugation of 1 L cultures. Ammonium sulfate precipitation (85% saturation) was carried out at 4 °C. After centrifugation, the precipitate was resuspended in succinate buffer (10 mM, pH 4.3) and dialyzed overnight against the same buffer (membrane cutoff of 10 kDa). The sample was applied to a cation-exchange resin (Macro-Prep High S, Bio-Rad, Hercules, CA, USA). After loading the sample into the column, extensive washing was performed to elute unwanted proteins. The enzyme was then eluted with a NaCl solution (150 mM). Fractions showing enzymatic activity with 2,2′-azino-bis(3-ethylbenzothiazoline-6-sulfonic acid (ABTS) (see Section 3.2.4) were pooled and concentrated in a Stirred Ultrafiltration Cell (AMICON, Millipore, Darmstadt, Germany) using a 10 kDa cutoff membrane. During ultrafiltration, the buffer was exchanged to TRIS (10 mM, pH 7.0). Further purification was achieved by anion-exchange chromatography (Macro-Prep High Q, Bio-Rad, Hercules, CA, USA). Once the sample was applied, the column was extensively washed with TRIS buffer before elution with a NaCl solution (120 mM). Enzyme active fractions were pooled and concentrated as the previous step, exchanging to potassium phosphate buffer (50 mM, pH 7.0). Specific activity and Reinheitszahl value (Rz, the absorbance ratio between 420 nm (Soret band) and 280 nm) were used as criteria for purity. For the subsequent kinetic analysis with the purified enzymes, routine protein concentration was determined with the Bradford method using bovine serum albumin as standard. 

#### 3.2.4. Enzyme Activity Assays

For all assays, the initial reaction rate was used to calculate the enzymatic units (the amount of enzyme that generates 1 μmol of product per minute). All measurements were performed in triplicate in an Agilent 8453 UV-Visible Spectroscopy System (Santa Clara, CA, USA).

Peroxidase activity. The activity with ABTS was monitored by the appearance of the stable one-electron-oxidized cation radical at 418 nm (molar extinction coefficient: 36,000 M^−1^ cm^−1^) [37]. The reaction mixture contained H_2_O_2_ (2 mM) and ABTS (0.4 mM) in a buffer mixture of sodium citrate and sodium phosphate (100 mM, adjusted to pH 4.4 with NaOH) [25]. 

Peroxygenase activity. The aromatic ether 5-nitro-1,3-benzodioxole (NBD) was used as a model substrate for specific peroxygenase activity [38]. The increase in absorbance of the product 4-nitrocatechol (molar extinction coefficient: 9700 M^−1^ cm^−1^) was followed at 425 nm in a reaction mixture containing potassium phosphate buffer (50 mM, pH 7.0), H_2_O_2_ (1 mM), and NBD (5 mM). The reaction mixture contained acetonitrile (20% *v*/*v*) for NBD solubilization. For naphthalene hydroxylation activity, the formation of the main product (1-naphthol) was followed at 303 nm (molar extinction coefficient: 2010 M^−1^ cm^−1^) [39]. The reaction contained naphthalene (2 mM) and H_2_O_2_ (1 mM) in potassium phosphate buffer (50 mM, pH 7.0) with acetonitrile (20% *v*/*v*). All measurements were performed in triplicate. 

Catalase activity. H_2_O_2_ conversion was monitored by the decrease in absorbance at 240 nm (molar extinction coefficient: 43.6 M^−1^ cm^−1^) [40]. The reaction mixture contained potassium phosphate buffer (50 mM, pH 7.0) and H_2_O_2_ (10 mM). The reaction was started by adding the enzyme (400 nM). All measurements were performed in triplicate.

#### 3.2.5. Determination of Kinetic Parameters

The kinetic parameters were determined for PaDa-I and its variants using ABTS, NBD, and naphthalene. The reaction mixtures were as mentioned in the previous section. Initial rate (usually the first 20 s of reaction, when the increase in absorbance was linear) at different substrate concentrations was measured. The substrate concentration range was as follows: for ABTS, the concentration range was 20–400 μM; for NBD, 100–1000 μM; and for naphthalene, 100–2000 μM. Typical enzyme concentration used for these determinations was as follows: 10 nM for ABTS; 30 nM for NBD and naphthalene. The data were adjusted to the Michaelis–Menten model using PRISM, in order to calculate the kinetic parameters k_cat_ and Km (Figures S2–S4). All measurements were performed in triplicate.

#### 3.2.6. Determination of the Total Turnover Number

The total turnover number (TTN) is the number of moles of converted substrate (or moles of generated product) per mole of enzyme during its operational lifetime, independent of time. In a typical experiment, substrate was monitored after the addition of enzyme and H_2_O_2_. Once substrate concentration remained constant (i.e., no more substrate conversion was observed), H_2_O_2_ (1 mM) was added to assess if substrate conversion was not limited by H_2_O_2_ and that the reaction had stopped due to enzyme inactivation. TTN was calculated as the amount of converted substrate (or generated product) divided by the amount of initial enzyme added to the reaction. A small amount of enzyme was used to convert just a fraction of the substrate and to ensure a uniform reaction rate during substrate conversion. Substrate conversion or product formation was measured as described below.

TTN was determined in a reaction containing the substrate (naphthalene: 2 mM, anthracene and phenanthrene: 250 μM) and H_2_O_2_ (1 mM) in potassium phosphate buffer (50 mM, pH 7.0) with acetonitrile (20% *v*/*v*). The reaction was initiated by the addition of enzyme (10 nM). Samples were taken at specific times and diluted with 1 volume of acetonitrile (ACN). PAH conversion was followed by reverse-phase HPLC in an Agilent 1100 Series equipment (Santa Clara, CA, USA) with a Zorbax Eclipse XDB-C18 column (4.6 × 150 mm, 5 μm) using the following ACN-water gradient in %*v*/*v*: ACN:H_2_O 30:70 for 3 min, 7 min to reach ACN:H_2_O 90:10, and then 5 min at 90:10. The flow was 0.4 mL min^−1^. The absorbance of the substrate was monitored at 250 nm and the area under the curve was used to quantify the remaining substrate. All measurements were performed in triplicate. A one-way ANOVA and several *t*-tests, two-sample assuming equal variances (*α* = 0.05), were performed to determine if there was a statistically significant difference between the averages of the TTN for each substrate and protein variant. 

#### 3.2.7. Stability of Enzyme Variants in Hydrogen Peroxide

Each enzyme variant (12 nM) was incubated in potassium phosphate buffer (50 mM, pH 7.0) and H_2_O_2_ (1 mM) at 25 °C. Aliquots were periodically withdrawn and the residual enzymatic activity was measured with the ABTS activity assay described in Section 3.2.4. All measurements were performed in triplicate. Data were fitted to a first-order inactivation model to calculate *k_inact_*.

## 4. Conclusions

In this work, we aimed to elucidate the role of bulky residues in the heme access channel. PaDa-I, a laboratory-evolved variant of UPO from *Agrocybe aegerita*, was used as model enzyme. The role of two amino acid residues, Phe76 and Phe191, was explored through site-directed mutagenesis and computational tools such as molecular dynamics (MD). According to MD simulations, we found that the replacement of Phe76 and Phe191 with less bulky residues (Ala or Leu) allowed a greater mobility in several residues of a loop and an alpha helix involved in shaping the heme access channel, which apparently led to a modified topography. This could result in a less productive interaction with the substrate, which explains the lower catalytic efficiency observed experimentally for the double mutants with peroxygenase substrates (naphthalene and NBD), which need to approach the vicinity of the activated heme group in order for the oxygen transfer to efficiently occur. Phe residues in positions 76 and 191 appear to be conserved in the subfamily I of peroxygenases, which could be related to enzyme specificity.

## Figures and Tables

**Table 1:** Kinetic parameters of PaDa-I mutants for the oxidation of standard substrates (5-nitro-1,3-benzodioxole (NBD) and 2,2′-azino-bis(3-ethylbenzothiazoline-6-sulfonic acid (ABTS)) and naphthalene.

Substrate	Kinetic Parameter *	PaDaI	F76A	F191A	F76L	F76A/F191A	F76L/F191A
NBD	k_cat_	286 ± 18	356 ± 30	279 ± 18	454 ± 22	258 ± 15	332 ± 33
	Km	736 ± 87	1386 ± 176	525 ± 73	890 ± 81	1389 ± 119	1552 ± 155
	k_cat_ /Km	3.89 × 10^5^	2.57 × 10^5^	5.31 × 10^5^	5.10 × 10^5^	1.86 × 10^5^	2.14 × 10^5^
Naphthalene	k_cat_	308 ± 25	305 ± 13	512 ± 28	341 ± 10	327 ± 17	485 ± 39
	Km	289 ± 23	544 ± 58	740 ± 41	466 ± 36	627 ± 78	486 ± 39
	k_cat_ /Km	1.07 × 10^6^	5.61 × 10^5^	6.92 × 10^5^	7.33 × 10^5^	5.22 × 10^5^	9.98 × 10^5^
ABTS	k_cat_	1620 ± 67	751 ± 77	1039 ± 33	1122 ± 49	702 ± 15	926 ± 65
	Km	186 ± 15	298 ± 51	93 ± 8	80 ± 9	136 ± 7	55 ± 12
	k_cat_ / Km	8.71 × 10^6^	2.52 × 10^6^	1.12 × 10^7^	1.40 × 10^7^	5.16 × 10^6^	1.68 × 10^7^

* Units: k_cat,_ s^−1^; Km, μM; k_ca*t*_/Km, M^−1^ s^−1^.

**Table 2:** Pseudo-first-order inactivation constants of PaDa-I and mutants F76A, F191A, F76L, F76A/F191A, and F76L/F191A when incubated in H_2_O_2_, and their catalase activity.

Variant	*k_inact_* (min^−1^)	t_1/2_ (min)	Catalase Specific Activity (U mg^−1^)
PaDa-I	0.181 ± 0.014	3.8 ± 0.3	704 ± 4
F76A	0.368 ± 0.048	1.9 ± 0.3	517 ± 29
F191A	0.204 ± 0.012	3.4 ± 0.2	686 ± 32
F76L	0.322 ± 0.062	2.2 ± 0.4	518 ± 10
F76A/F191A	0.250 ± 0.014	2.8 ± 0.2	595 ± 7
F76L/F191A	0.267 ± 0.012	2.6 ± 0.1	1349 ± 234

## Supplementary Materials

Supplementary materials can be found at https://www.mdpi.com/1422-0067/21/16/5734/s1. Figure S1: SDS-PAGE of selected variants with a Rz of 2.0 to corroborate their purity. A total of 5 µg of protein were loaded on each well. The gel was stained with silver nitrate. The molecular weight marker was Precision Plus Protein Standard (Biorad); Figure S2: Michaelis-Menten plots for the enzyme variants for the substrate ABTS. All model fits had a R^2^>0.95; Figure S3: Michaelis-Menten plots for the enzyme variants for the substrate NBD. All model fits had a R^2^>0.95; Figure S4: Michaelis-Menten plots for the enzyme variants for the substrate naphthalene. All model fits had a R^2^>0.95; Figure S5: Inactivation kinetics of PaDa-I and its variants in the presence of hydrogen peroxide; Figure S6: Root mean square deviation (RMSD) of the whole protein for PaDa-I and F76A, F191A, F76L, F76A/F191A and F76L/F191A variants during a 50 ns molecular dynamics simulation. All the atoms were considered in the calculation; Figure S7: Root mean square deviation (RMSD) of the heme channel for PaDa-I and F76A, F191A, F76L, F76A/F191A and F76L/F191A variants during a 50 ns molecular dynamics simulation. The heme channel is delimited by the following residues: Phe69, Asp70, Gln72, Ala73, Phe76, Ala77, Thr78, Ala80, Ala81, Phe121, Phe188, Arg189, Phe191, Thr192, Glu196, Phe199, Leu203, Ser240, Phe274, Ala316, Ala317. The RMSD value was calculated over the alpha carbon of each amino acid residue: Figure S8: Reorientations of the network of residues 76, 280, 277, 188 and 191 due to the mutations of Phe76 and Phe191 give in some cases flexibility of the loop that contains Met280 and/or the alpha-helix that contains the residue 191. In the case of the double mutant F76L/F191A, the reorientation of this network favours a close packaging that does not allow flexibility of the mentioned loop and helix. The heme group is shown in red sticks as reference; Figure S9: Graphical representation of the shapes of the volume conforming the heme channel (in blue) with respect to the heme group (red spheres); Figure S10: Frequency distribution of the dihedral angle variations of residues Phe76 (panels A and B) and Phe191 (panels C and D) during the last 40 ns of MD simulation; Figure S11: Graphical representation of a segment of the multiple sequence alignment of subfamily I (A and C) and subfamily V (B and D), according to the UPO classification in reference 13. Subfamily I is represented by sequences of 12 putative UPOs and AaeUPO, whereas subfamily V comprises sequences from 8 putative UPOs. The residues numbering corresponds to AaeUPO. The image was created with WebLogo; Figure S12: Upper view of the substrate access channel of PaDa-I (PDB: 5OXU). Heme group is represented as red spheres. The N-glycosylation sites are shown in orange sticks (Asn residues 11, 141, 161, 182 and 286). Special protonated residues according to PropKa (Asp85, His82, His118, His251) are also shown in sticks and are located away from the heme channel; Table S1: Residues with increased (+) or decreased (-) mobility with respect to PaDa-I, according to ΔRMSF. Residues with changes in mobility that occur in more than one enzyme variant are highlighted in orange (+ mobility) or blue (- mobility); Table S2: Oligonucleotide primer sequences used in the generation of PaDa-I mutants. The mutated codon is shown bold and underlined.

## References

[1] Ullrich R., Nüske J., Scheibner K., Spantzel J., Hofrichter M. (2004). Novel haloperoxidase from the agaric basidiomycete *Agrocybe aegerita* oxidizes aryl alcohols and aldehydes. Appl. Environ. Microbiol..

[2] Ullrich R., Hofrichter M. (2005). The haloperoxidase of the agaric fungus *Agrocybe aegerita* hydroxylates toluene and naphthalene. FEBS Lett..

[3] Hofrichter M., Ullrich R. (2014). Oxidations catalyzed by fungal peroxygenases. Curr. Opin. Chem. Biol..

[4] Kluge M., Ullrich R., Dolge C., Scheibner K., Hofrichter M. (2009). Hydroxylation of naphthalene by aromatic peroxygenase from *Agrocybe aegerita* proceeds via oxygen transfer from H_2_O_2_ and intermediary epoxidation. Appl. Microbiol. Biotechnol..

[5] Aranda E., Ullrich R., Hofrichter M. (2010). Conversion of polycyclic aromatic hydrocarbons, methyl naphthalenes and dibenzofuran by two fungal peroxygenases. Biodegradation.

[6] Peter S., Kinne M., Wang X., Ullrich R., Kayser G., Groves J.T., Hofrichter M. (2011). Selective hydroxylation of alkanes by an extracellular fungal peroxygenase. FEBS J..

[7] Kinne M., Poraj-Kobielska M., Ralph S.A., Ullrich R., Hofrichter M., Hammel K.E. (2009). Oxidative Cleavage of Diverse Ethers by an Extracellular Fungal Peroxygenase. J. Biol. Chem..

[8] Babot E.D., del Río J.C., Cañellas M., Sancho F., Lucas F., Guallar V., Kalum L., Lund H., Gröbe G., Scheibner K. (2015). Steroid Hydroxylation by Basidiomycete Peroxygenases: A Combined Experimental and Computational Study. Appl. Environ. Microbiol..

[9] Hofrichter M., Ullrich R. (2006). Heme-thiolate haloperoxidases: Versatile biocatalysts with biotechnological and environmental significance. Appl. Microbiol. Biotechnol..

[10] Ahn D.H., Ullrich R., Benndorf D., Svatos A., Muck A., Hofrichter M. (2007). The Coprophilous Mushroom *Coprinus radians* Secretes a Haloperoxidase That Catalyzes Aromatic Peroxygenation. Appl. Environ. Microbiol..

[11] Gröbe G., Ullrich R., Pecyna M.J., Kapturska D., Friedrich S., Hofrichter M., Scheibner K. (2011). High-yield production of aromatic peroxygenase by the agaric fungus *Marasmius rotula*. AMB Express.

[12] Kiebist J., Schmidtke K., Zimmermann J., Kellner H., Jehmlich N., Ullrich R., Zänder D., Hofrichter M., Scheibner K. (2017). A Peroxygenase from *Chaetomium globosum* Catalyzes the Selective Oxygenation of Testosterone. ChemBioChem.

[13] Faiza M., Huang S., Lan D., Wang Y. (2019). New insights on unspecific peroxygenases: Superfamily reclassification and evolution. BMC Evol. Biol..

[14] Hofrichter M., Kellner H., Herzog R., Karich A., Liers C., Scheibner K., Kimani V.W., Ullrich R., Nevalainen H. (2020). Fungal peroxygenases: A phylogenetically old superfamily of heme enzymes with promiscuity for oxygen transfer reactions. Grand Challenges in Fungal Biotechnology. Grand Challenges in Biology and Biotechnology.

[15] Piontek K., Strittmatter E., Ullrich R., Gröbe G., Pecyna M.J., Kluger M., Scheibner K., Hofrichter M., Plattner D.A. (2013). Structural Basis of Substrate Conversion in a New Aromatic Peroxygenase: Cytochrome P450 functionality with benefits. J. Biol. Chem..

[16] Ramirez-Escudero M., Molina-Espeja P., Gomez de Santos P., Hofrichter M., Sanz-Aparicio J., Alcalde M. (2018). Structural Insights into the Substrate Promiscuity of a Laboratory-Evolved Peroxygenase. ACS Chem. Biol..

[17] Sundaramoorthy M., Terner J., Poulos T.L. (1995). The crystal structure of chloroperoxidase: A heme peroxidase-cytochrome P450 functional hybrid. Structure.

[18] Olmedo A., del Rio J.C., Kiebist J., Ullrich R., Hofrichter M., Scheibner K., Martinez A.T., Gutierrez A. (2017). From Alkanes to Carboxylic Acids: Terminal Oxygenation by a Fungal Peroxygenase. Chem. Eur. J..

[19] Peter S., Kinne M., Ullrich R., Kayser G., Hofrichter M. (2013). Epoxidation of linear, branched and cyclic alkenes catalyzed by unspecific peroxygenase. Enz. Microb. Technol..

[20] Ayala M., Pickard M.A., Vazquez-Duhalt R. (2008). Fungal Enzymes for Environmental Purposes, a Molecular Biology Challenge. J. Mol. Microbiol. Biotechnol..

[21] Wang Y., Lan D., Durrani R., Hollmann F. (2017). Peroxygenases en route to becoming dream catalysts. What are the opportunities and challenges?. Curr. Opin. Chem. Biol..

[22] Garcia-Guevara F., Avelar M., Ayala M., Segovia L. (2015). Computational Tools Applied to Enzyme Design—A review. Biocatalysis.

[23] Molina-Espeja P., Canellas M., Plou F.J., Hofrichter M., Lucas F., Guallar V., Alcalde M. (2016). Synthesis of 1-Naphthol by a Natural Peroxygenase Engineered by Directed Evolution. ChemBioChem.

[24] Gomez de Santos P., Canellas M., Tieves F., Younes S.H.H., Molina-Espeja P., Hofrichter M., Hollmann F., Guallar V., Alcalde M. (2018). Selective Synthesis of the Human Drug Metabolite 5′-Hydroxypropranolol by an Evolved Self-Sufficient Peroxygenase. ACS Catal..

[25] Molina-Espeja P., Garcia-Ruiz E., Gonzalez-Perez D., Ullrich R., Hofrichter M., Alcalde M. (2014). Directed Evolution of Unspecific Peroxygenase from Agrocybe aegerita. Appl. Environ. Microbiol..

[26] Samanta S.K., Singh O.V., Jain R.K. (2002). Polycyclic aromatic hydrocarbons: Environmental pollution and bioremediation. Trends Biotechnol..

[27] Ortiz de Montellano P.R., Torres E., Ayala M. (2010). Catalytic Mechanisms of Heme Peroxidases. Biocatalysis Based on Heme Peroxidases.

[28] Polizzi K.M., Bommarius A.S., Broering J.M., Chaparro-Riggers J.F. (2007). Stability of biocatalysts. Curr. Op. Chem. Biol..

[29] Valderrama B., Ayala M., Vazquez-Duhalt R. (2002). Suicide Inactivation of Peroxidases and the Challenge of Engineering More Robust Enzymes. Chem. Biol..

[30] Ayala M., Batista C.V., Vazquez-Duhalt R. (2011). Heme destruction, the main molecular event during the peroxide-mediated inactivation of chloroperoxidase from *Caldariomyces fumago*. J. Biol. Inorg. Chem..

[31] Karich A., Scheibner K., Ullrich R., Hofrichter M. (2016). Exploring the catalase activity of unspecific peroxygenases and the mechanism of peroxide-dependent heme destruction. J. Mol. Catal. B Enzym..

[32] (2014). Schrödinger Release 2014-2: Maestro, Schrödinger.

[33] Bowers K.J., Chow E., Xu H., Dror R.O., Eastwood M.P., Gregersen B.A., Klepeis J.L., Kolossvary I., Moraes M.A., Sacerdoti F.D. Scalable algorithms for molecular dynamics simulations on commodity clusters. Proceedings of the ACM/IEEE Conference on Supercomputing (SC06).

[34] Humphrey W., Dalke A., Schulten K. (1996). VMD: Visual molecular dynamics. J. Mol. Graph..

[35] Wagner J.R., Sørensen J., Hensley N., Wong C., Zhu C., Perison T., Amaro R.E. (2017). POVME 3.0: Software for Mapping Binding Pocket Flexibility. J. Chem. Theory Comp..

[36] Gonzalez-Perez D., Garcia-Ruiz E., Alcalde M. (2012). *Saccharomyces cerevisiae* in directed evolution. An efficient tool to improve enzymes. Bioeng. Bugs.

[37] Childs R.E., Bardsley W.G. (1975). The steady-state kinetics of peroxidase with 2,2′-azino-di-(3-ethyl-benzthiazoline-6-sulphonic acid) as chromogen. Biochem. J..

[38] Poraj-Kobielska M., Kinne M., Ullrich R., Scheibner K., Hofrichter M. (2012). A spectrophotometric assay for the detection of fungal peroxygenases. Analyt. Biochem..

[39] Kluge M.G., Ullrich R., Scheibner K., Hofrichter M. (2007). Spectrophotometric assay for detection of aromatic hydroxylation catalyzed by fungal haloperoxidase–peroxygenase. Appl. Microbiol. Biotechnol..

[40] Noble R.W., Gibson Q.H. (1970). The Reaction of Ferrous Horseradish Peroxidase with Hydrogen Peroxide. J. Biol. Chem..

