# Supplementary material for: Exploring the Role of Phenylalanine Residues in Modulating the Flexibility and Topography of the Active Site in the Peroxygenase Variant PaDa-I
Source: Int J Mol Sci. 2020 Aug 10;21(16):5734. doi: 10.3390/ijms21165734 (PMC7460833; doi:10.3390/ijms21165734)
Supplement: Supplementary file 1 [file ijms-21-05734-s001.pdf]

Supplementary Materials

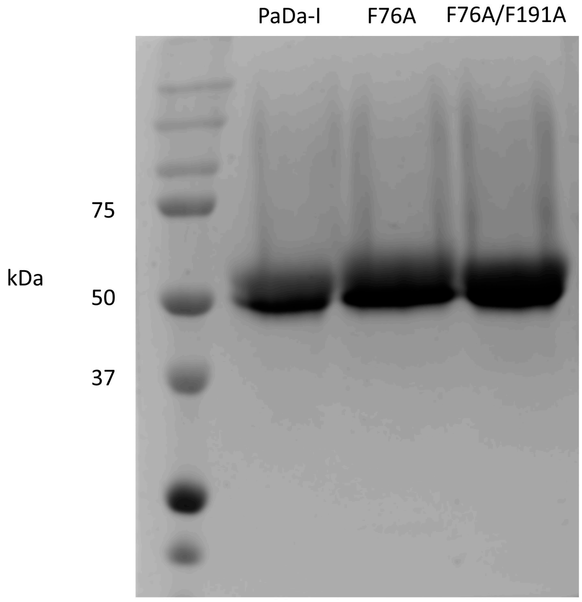

Figure S1. SDS-PAGE of selected variants with an Rz of 2.0 to corroborate their purity. A total of 5 µg of protein were loaded on each well. The gel was stained with silver nitrate. The molecular weight marker was Precision Plus Protein Standard (Bio-Rad).

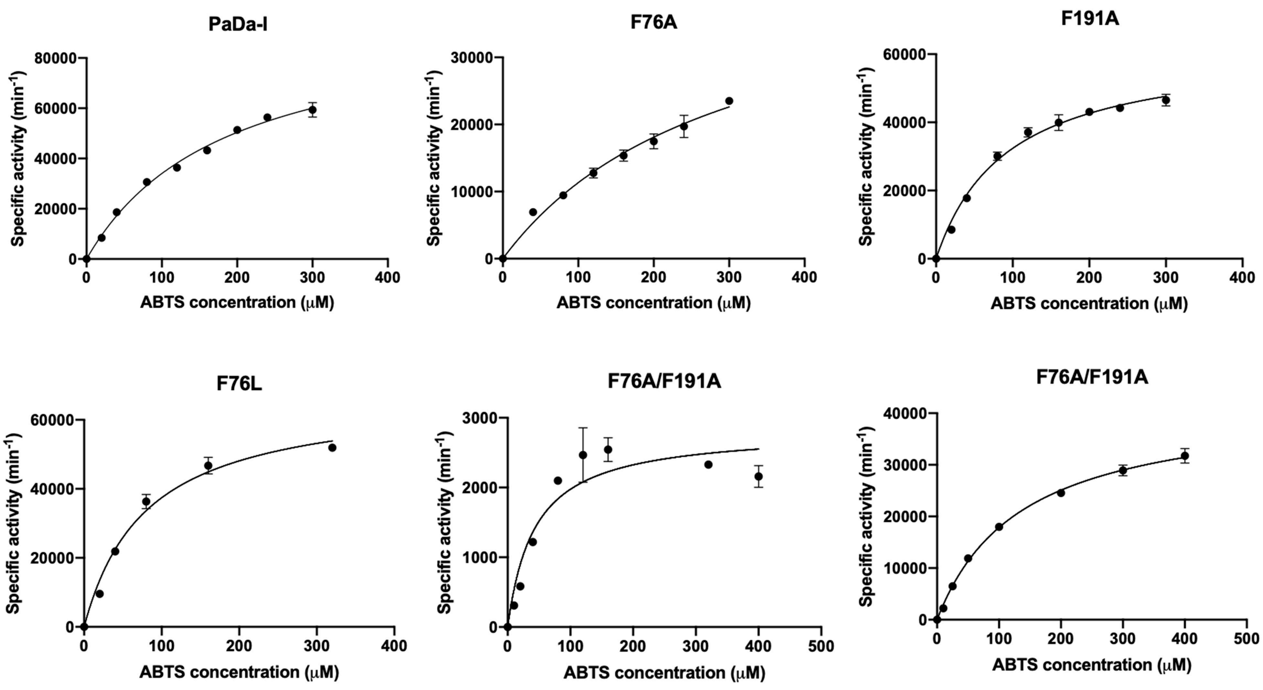

Figure S2. Michaelis–Menten plots for the enzyme variants for the substrate ABTS. All model fits had a R^2>0.95.

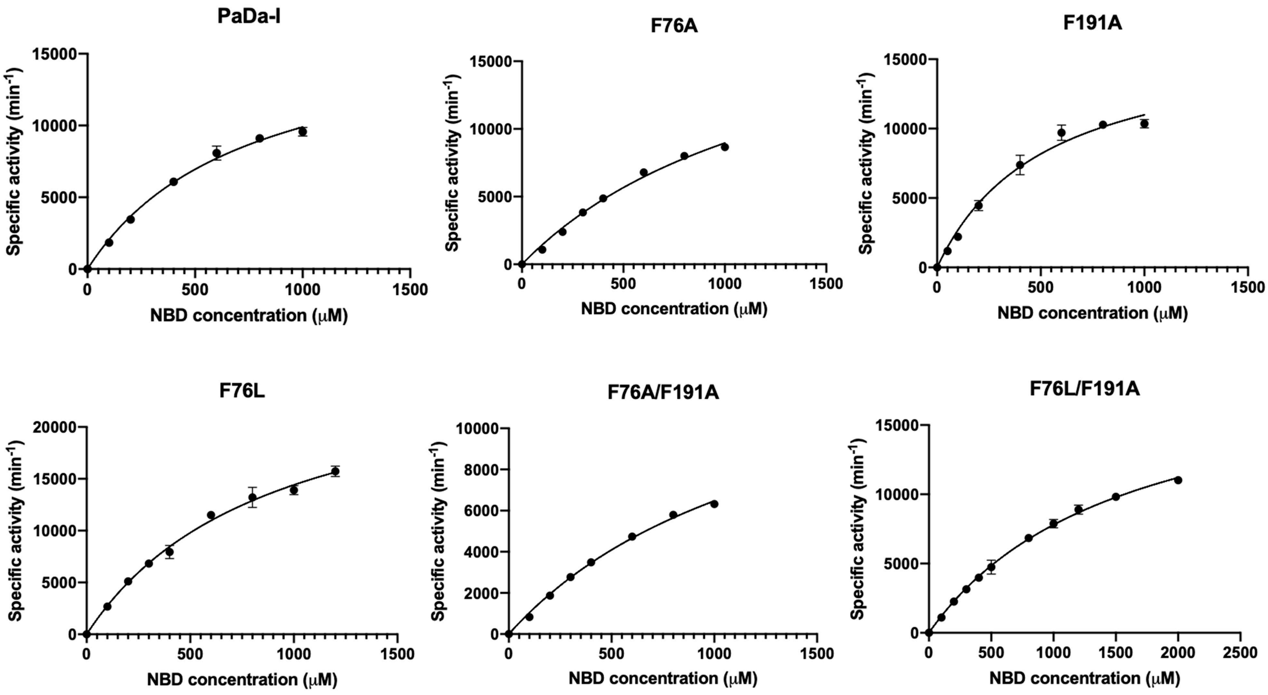

Figure S3. Michaelis–Menten plots for the enzyme variants for the substrate NBD. All model fits had a R^2>0.95.

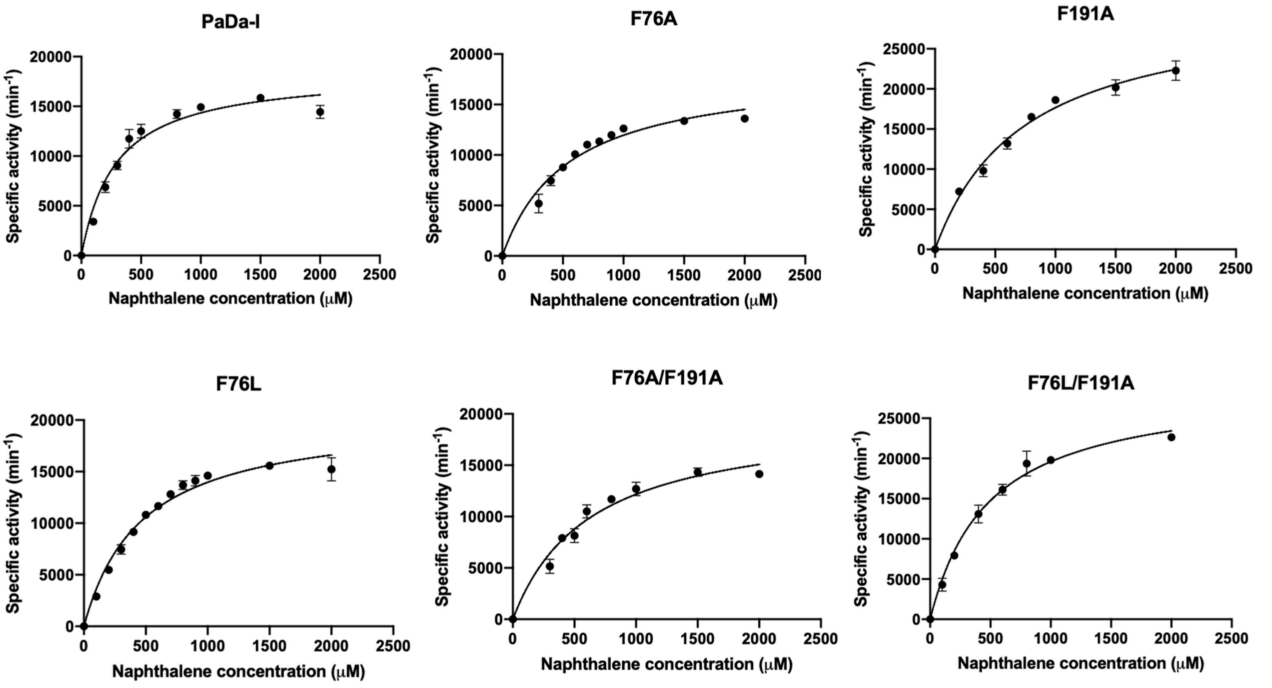

Figure S4. Michaelis–Menten plots for the enzyme variants for the substrate naphthalene. All model fits had a $R^2>0.95$.

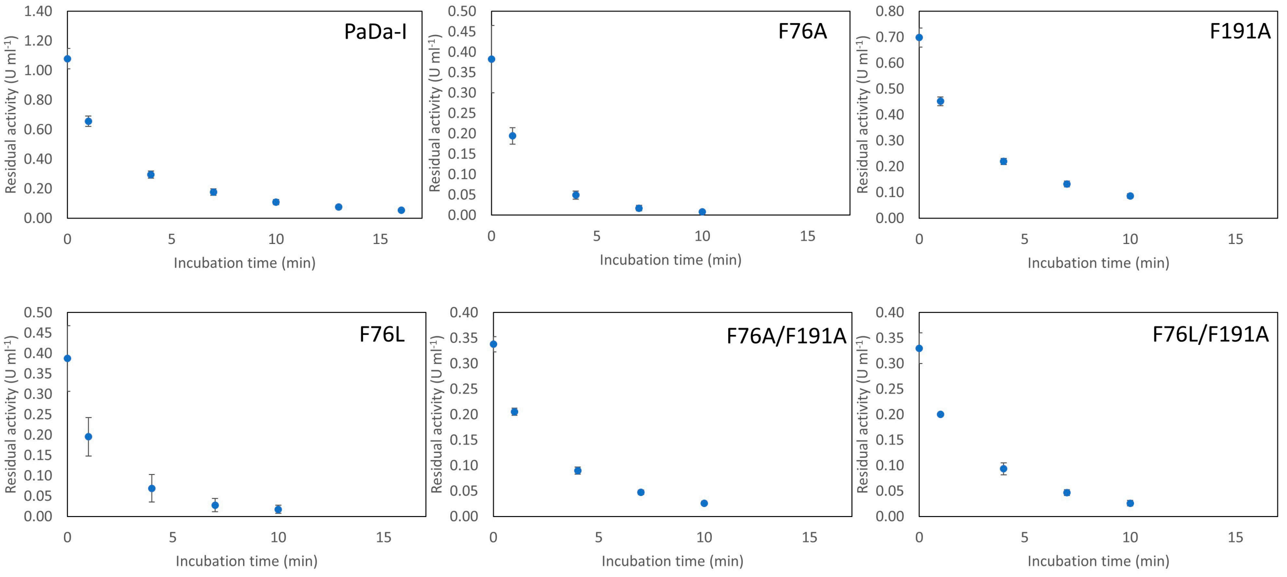

Figure S5. Inactivation kinetics of PaDa-I and its variants in the presence of hydrogen peroxide.

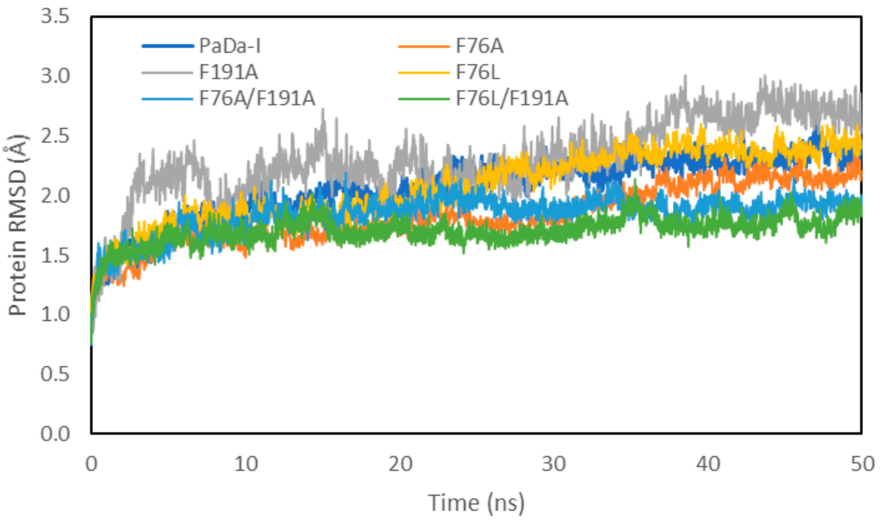

Figure S6. Root mean square deviation (RMSD) of the whole protein for PaDa-I and F76A, F191A, F76L, F76A/F191A, and F76L/F191A variants during a 50 ns molecular dynamics simulation. All the atoms were considered in the calculation.

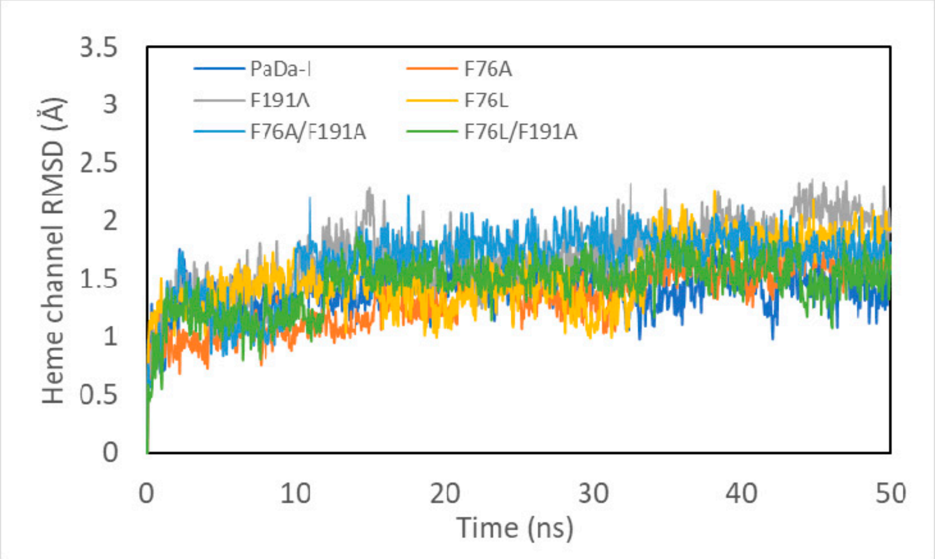

Figure S7. Root mean square deviation (RMSD) of the heme channel for PaDa-I and F76A, F191A, F76L, F76A/F191A, and F76L/F191A variants during a 50 ns molecular dynamics simulation. The heme channel is delimited by the following residues: Phe69, Asp70, Gln72, Ala73, Phe76, Ala77, Thr78, Ala80, Ala81, Phe121, Phe188, Arg189, Phe191, Thr192, Glu196, Phe199, Leu203, Ser240, Phe274, Ala316, and Ala317. The RMSD value was calculated over the alpha carbon of each amino acid residue.

Table S1. Residues with increased (+) or decreased (-) mobility with respect to PaDa-I, according to ΔRMSF. Residues with changes in mobility that occur in more than one enzyme variant are highlighted in orange (+ mobility) or blue (- mobility).

F76A		F191A		F76L		F76A/F191A		F76L/F191A	
-	+	-	+	-	+	-	+	-	+
								Pro2	
								Gly3	
						Ala21			
								Ser44	
								His45	
Gly46								Gly46	
								Asp91	
						Gly123		Gly123	
Ser126									
Gly130		Gly130		Gly130		Gly130		Gly130	
				Asn137					
	Glu142								
		Leu162		Leu162					
		Ala165							
		Gly166							
						Asn180			
						Asn182			
								Phe183	
								Ser184	
			Asp187						
	Phe188		Phe188				Phe188		
			Arg189						
			Phe190						
			Ala191				Ala191		
			Thr192						
			Ala193		Ala193		Ala193		
			Tyr194		Tyr194				
					Gly195				
					Glu196				
					Thr197				
					Asp210				
					Asp211				
									Gly241
			Gly243				Gly243		
Val246									
	Pro252								
			Pro255						
			Thr270						
			Ser271						
			Ser272						
								Asp273	
			Thr276						
			Pro277						

			Cys278						
			Leu279						
	Met280		Met280						
Ala316									
	Gln321	Gln321		Gln321		Gln321		Gln321	
						Val322		Val322	
						Phe323			

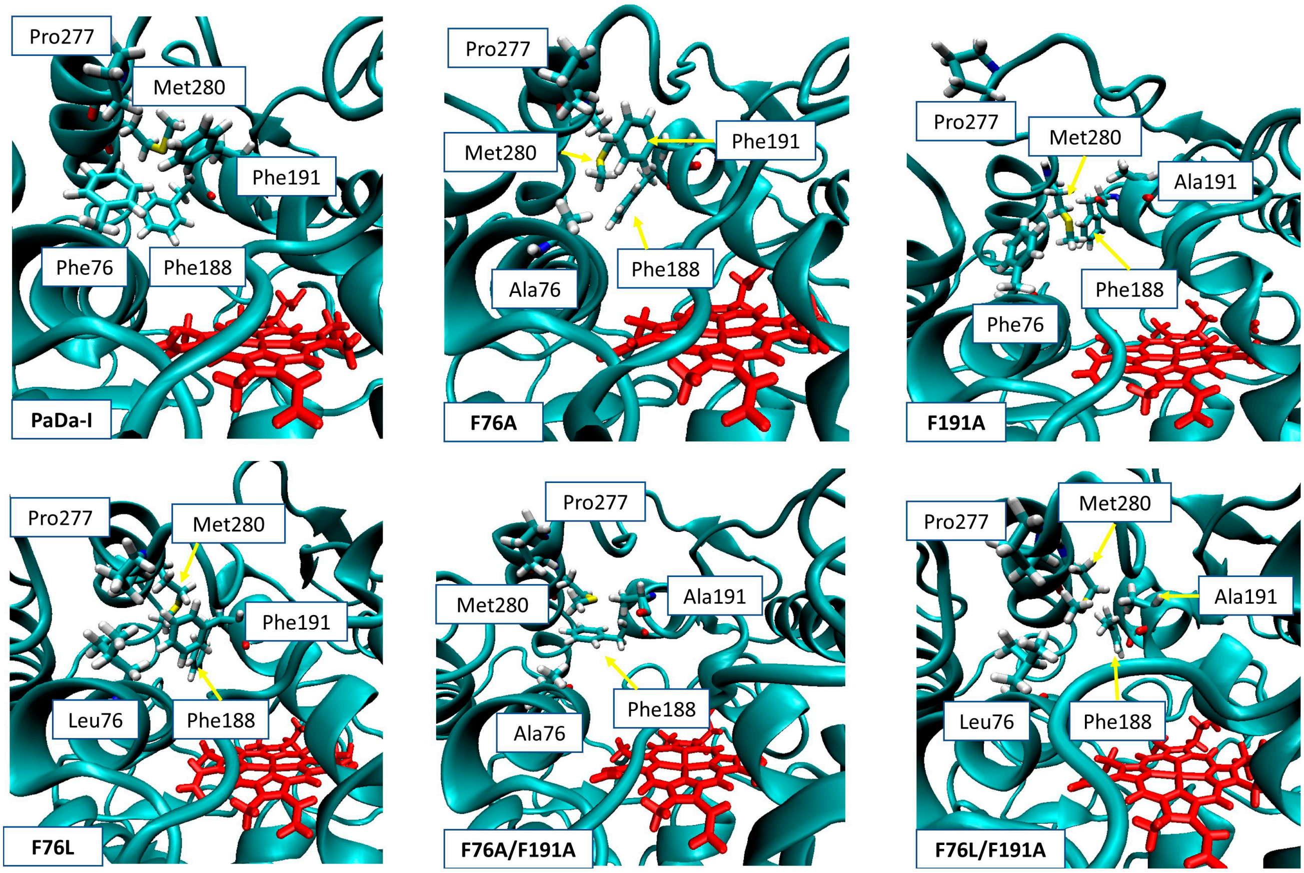

Figure S8. Reorientations of the network of residues 76, 280, 277, 188, and 191 due to the mutations of Phe76 and Phe191 give in some cases flexibility of the loop that contains Met280 and/or the alpha helix that contains the residue 191. In the case of the double mutant F76L/F191A, the reorientation of this network favors a close packaging that does not allow flexibility of the mentioned loop and helix. The heme group is shown in red sticks as reference.

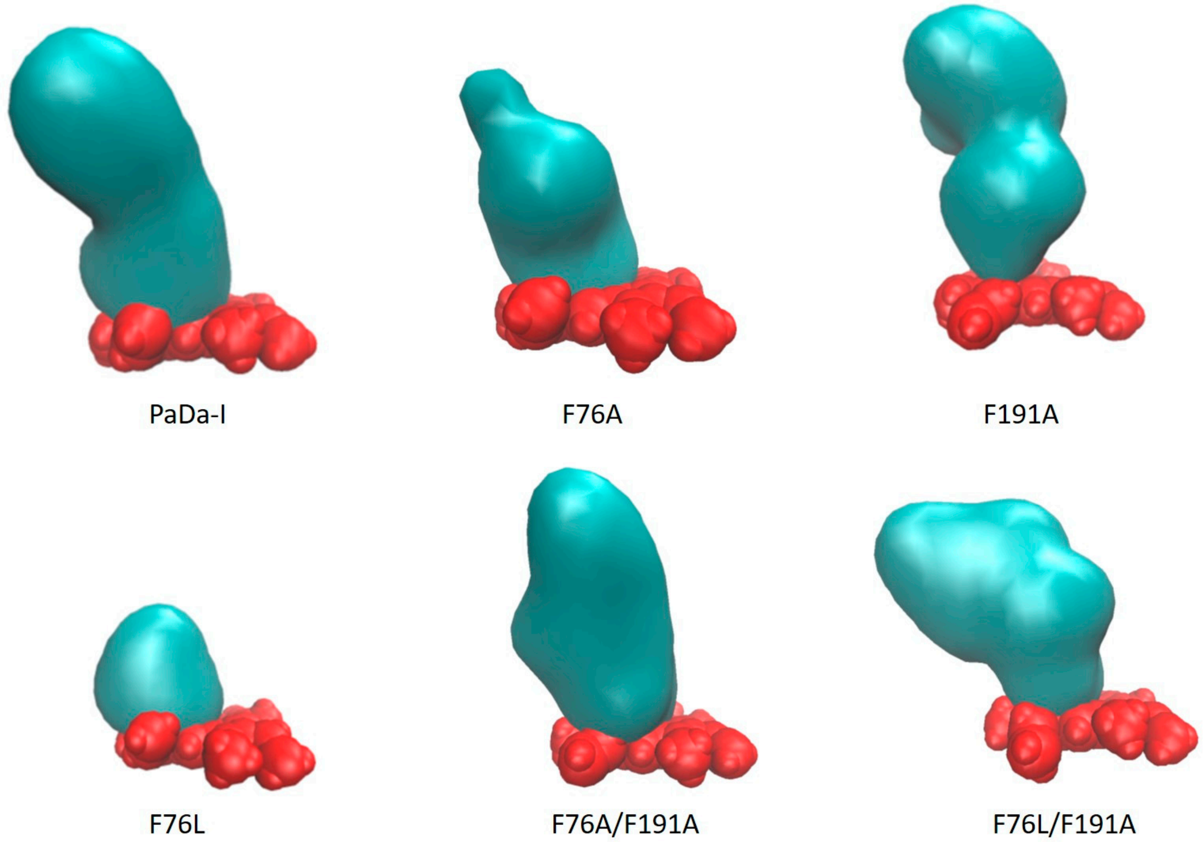

Figure S9. Graphical representation of the shapes of the volume conforming the heme channel (in blue) with respect to the heme group (red spheres).

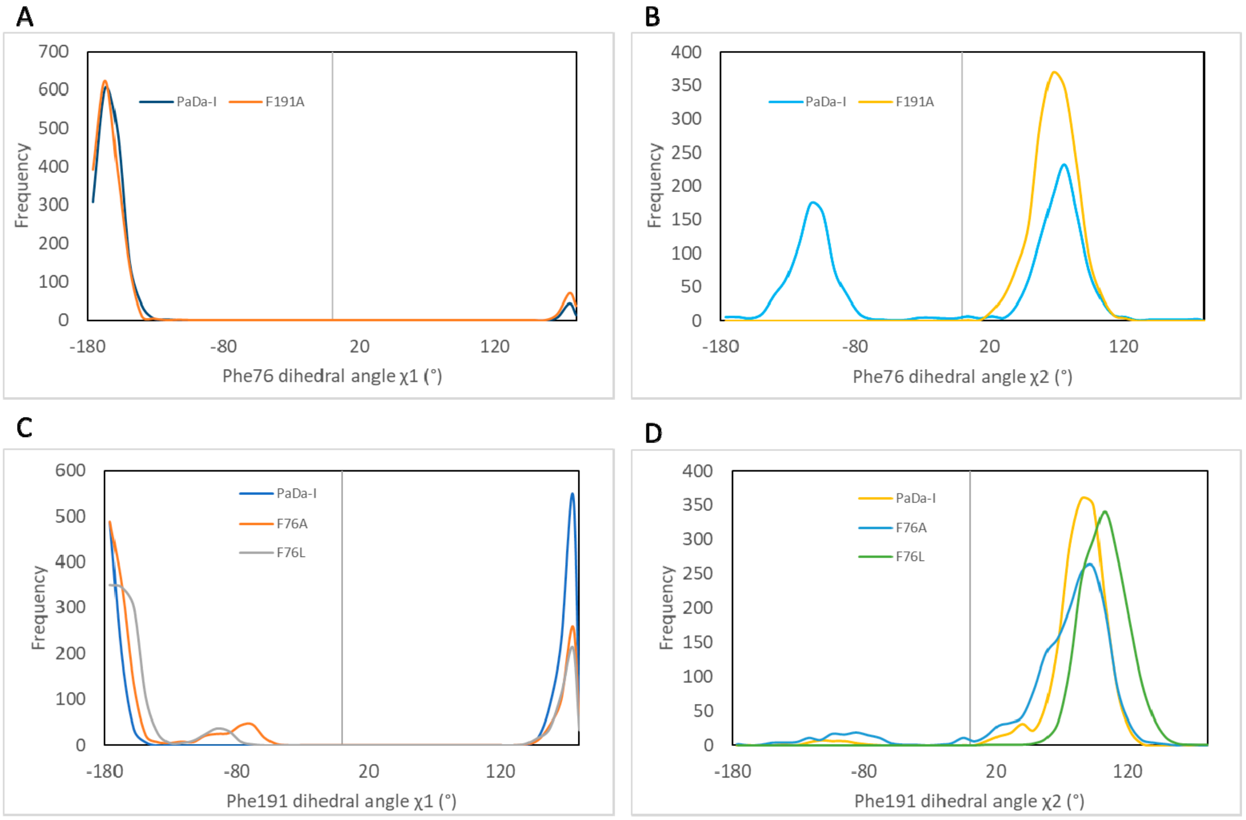

Figure S10. Frequency distribution of the dihedral angle variations of residues Phe76 (panels A and B) and Phe191 (panels C and D) during the last 40 ns of MD simulation.

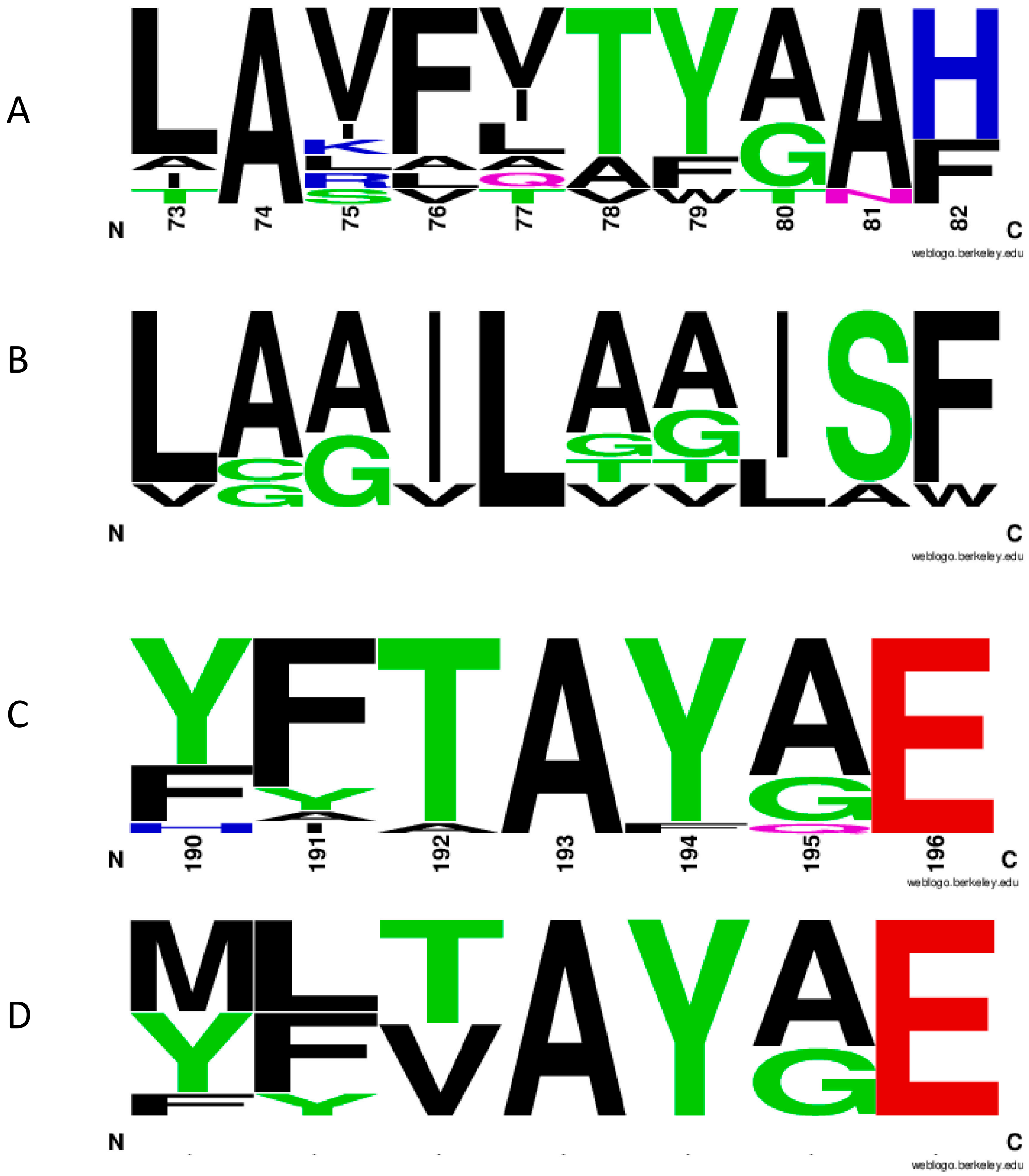

Figure S11. Graphical representation of a segment of the multiple sequence alignment of subfamily I (A and C) and subfamily V (B and D), according to the UPO classification in [13]. Subfamily I is represented by sequences of 12 putative UPOs and AaeUPO, whereas subfamily V comprises sequences from 8 putative UPOs. The residues' numbering corresponds to AaeUPO. The image was created with WebLogo [41].

Table S2. Oligonucleotide primer sequences used in the generation of PaDa-I mutants. The mutated codon is shown bold and underlined.

Primer name	Sequence (5'-3')
RMLN	CCT-CTA-TAC-TTT-AAC-GTC-AAG-G
RMLC	GGG-AGG-GCG-TGA-ATG-TAA-GC
f76a-Forward	C-GAC-AAT-CAA-GCC-GCA-ATC-***GCT-***GCC-ACA-TAT-GCG-GCC-CAC-C
f76a-Reverse	G-GTG-GGC-CGC-ATA-TGT-GGC-***AGC-***GAT-TGC-GGC-TTG-ATT-GTC-G
f191a-Forward	CC-TTT-GTT-GAC-TTT-AGG-TTC-***GCT-***ACT-GCT-TAC-GGC-GAG-ACC-ACC
f191a-Reverse	GGT-GGT-CTC-GCC-GTA-AGC-AGT-***AGC-***GAA-CCT-AAA-GTC-AAC-AAA-GG
f76l-Forward	C-GAC-AAT-CAA-GCC-GCA-ATC-***TTG***-GCC-ACA-TAT-GCG-GCC-CAC-C
f76l-Reverse	G-GTG-GGC-CGC-ATA-TGT-GGC-***CAA-***GAT-TGC-GGC-TTG-ATT-GTC-G

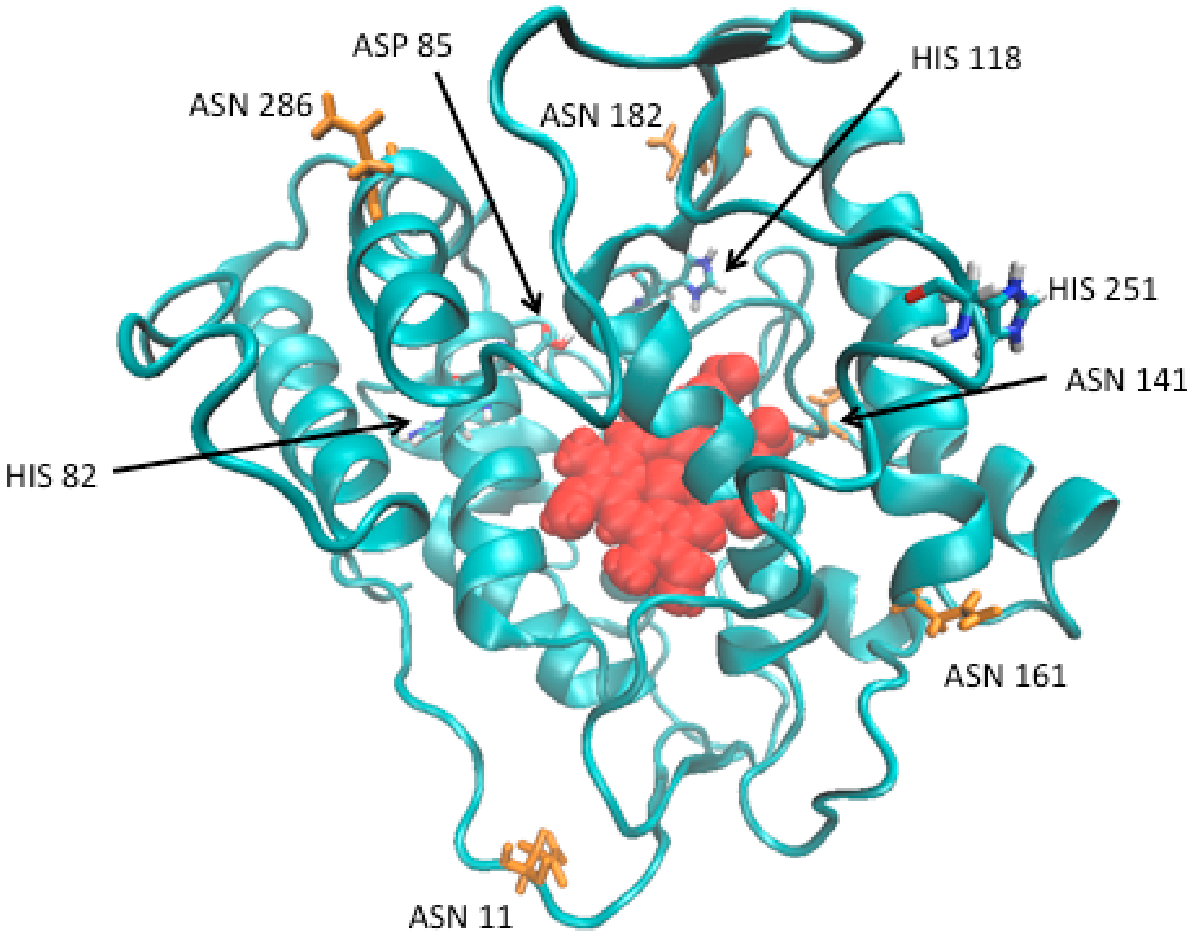

Figure S12. Upper view of the substrate access channel of PaDa-I (PDB: 5OXU). Heme group is represented as red spheres. The N-glycosylation sites are shown in orange sticks (Asn residues 11, 141, 161, 182, and 286). Special protonated residues according to PropKa (Asp85, His82, His118, and His251) are also shown in sticks and are located away from the heme channel.

References

[41] Crooks, G.E.; Hon, G.; Chandonia, J.M.; Brenner, S.E. WebLogo: a sequence logo generator. *Genome Res.* **2004**, *14*, 1188-1190.
